# Determinants of Oxygen Therapy in Childhood Pneumonia in a Resource-Constrained Region

**DOI:** 10.1155/2013/435976

**Published:** 2013-06-02

**Authors:** Bankole Peter Kuti, Samuel Ademola Adegoke, Benard E. Ebruke, Stephen Howie, Oyeku Akibu Oyelami, Martin Ota

**Affiliations:** ^1^Department of Paediatrics and Child Health, Obafemi Awolowo University, Ile-Ife, Osun State, Nigeria; ^2^Medical Research Council, Gambian Unit, Atlantic Boulevard, Fajara, P.O. Box 273, Banjul, Gambia

## Abstract

Childhood pneumonia is a leading cause of morbidity and mortality among underfives particularly in the resource-constraint part of the world. A high proportion of these deaths are due to lack of oxygen, thereby making oxygen administration a life-saving adjunctive when indicated. However, many primary health centres that manage most of the cases often lack the adequate manpower and facilities to decide which patient should be on oxygen therapy. Therefore, this study aimed to determine factors that predict hypoxaemia at presentation in children with severe pneumonia. Four hundred and twenty children aged from 2 to 59 months (40% infants) with severe pneumonia admitted to a health centre in rural Gambia were assessed at presentation. Eighty-one of them (19.30%) had hypoxaemia (oxygen saturation < 90%). Children aged 2–11 months, with grunting respiration, cyanosis, and head nodding, and those with cardiomegaly on chest radiograph were at higher risk of hypoxaemia (*P* < 0.05). Grunting respiration (OR = 5.210, 95% CI 2.287–7.482) and cyanosis (OR = 83.200, 95% CI 5.248–355.111) were independent predictors of hypoxaemia in childhood pneumonia. We conclude that children that grunt and are centrally cyanosed should be preferentially commenced on oxygen therapy even when there is no facility to confirm hypoxaemia.

## 1. Introduction

Pneumonia is the inflammation of the lung parenchyma mostly caused by infectious agents in children [1]. These infectious agents are mainly bacteria and viruses. The inflammatory changes in the lungs impair effective gas exchange leading to its various clinical manifestations [1]. 

Childhood pneumonia is a leading cause of morbidity and mortality in under-fives especially in developing countries [2, 3]. It is a major cause of hospital admission and death being responsible for approximately one out of every five deaths among under-fives globally [2, 3]. The World Health Organization (WHO) estimated that about 156 million new cases of pneumonia occur in under-five children each year worldwide, of which 151 million episodes (>90 percent) occur in the developing world [2]. This translates to an incidence in under-five children estimated to be 0.29 episodes per child-year (with an interquartile range 0.21–0.71) in developing countries and 0.05 episodes per child-year in developed countries [2]. Most cases of pneumonia occur in Asia and sub-Saharan Africa with India (43 million), China (21 million), Indonesia and Nigeria (6 million each), taking the lion's share of the burden [2, 3].

A recent report from Gambia estimated that 13.4 episodes of severe pneumonia per 1000 child-years occurred in children receiving vaccines against both Pneumococcus and *Haemophilus influenzae* type b [4]. Pneumonia is also the most important cause of death among under-five Gambian children accounting for 11 percent of paediatric admission and 7 percent of deaths from a tertiary centre in Gambia [5].

Mortality from childhood pneumonia usually results from inability to detect or recognise signs associated with severity [5–8]. One major contributor of mortality in pneumonia is hypoxaemia [9–12]. Hypoxaemia, defined as low arterial blood oxygen saturation less than 90% by pulse oximetry, correlates well with partial arterial oxygen pressure of less than 60 mmHg. This is the point at which precipitous decline in clinical status occur in ill patients. [13, 14]. Hypoxaemia results from impaired gas exchange due to the inflammation causing alveolar congestion, increased dead space, marked intrapulmonary shunting, and ventilation-perfusion mismatch [13, 14]. Airflow obstruction from respiratory tract secretions, respiratory muscle fatigue as well as reduced central respiratory centre response to hypoxia and hypercarbia also contribute to the hypoxaemic state [13, 14]. The case fatality of pneumonia has been reported to be inversely related to oxygen saturation of arterial blood [9–12]. The oxygen saturation has also been shown to predict outcome and even long-term morbidity and mortality in childhood pneumonia [15]. Delivering oxygen to hypoxaemic children therefore improves the outcome of childhood pneumonia [15].

Children with pneumonia in the resource-constrained regions of the world are mostly managed by primary health workers [16], who are often confused on when or who to administer oxygen, particularly in situations when demand outweighs supply [7]. Consequently, many workers have tried to determine the factors in childhood pneumonia that can predict the need for oxygen therapy [9–12]. Many of these works were done among children with acute lower respiratory tract infections including both severe and nonsevere forms and mostly in urban settings [9, 10]. There is therefore a need to be able to recognise factors at presentation that can predict the need for oxygen therapy among cohort of under-fives with severe and very severe pneumonia in a rural resource constraint setting where the burden of pneumonia is high.

## 2. Patients and Methods

The study was conducted between November 2010 and April 2011 as a prospective observational study of consecutive admission of children aged 2 to 59 months with severe and very severe pneumonia using the WHO criteria [8] at the paediatric ward of the Basse Major Health Centre, in rural Gambia. The health centre serves as a referral health facility for the 225 adjoining villages in Basse. Basse is located at 13°28′ North of the equator and longitude 14°36′ West of the meridian at sea level, with a mean temperature of 24°C (18–36°C), relative humidity of 41%, and annual rainfall of 876 mm [17].

The following were used as criteria for enrolment into the study: age 2–59 months, difficult or fast breathing with rate >50 or 40 cycles per minute for ages 2–11, and 12–59 months, respectively. In addition to fast or difficult breathing, patient must have at least one of the followings: lower chest in-drawing, central cyanosis, lethargy or altered sensorium and inability to feed or drink. Children who fulfilled above criteria were recruited as cases of severe and very severe pneumonia (clinical pneumonia) irrespective of findings on Chest radiograph. Patients with wheeze and cough lasting more than two weeks were excluded to reduce the chances of recruiting cases of bronchial asthma, bronchiolitis, and tuberculosis, respectively. History of the presenting illness, like cough, fever, fast/difficulty in breathing, chest pain, and inability to suck, drink, or feed, and the duration were documented. Associated illnesses like diarrhoea, vomiting, and convulsion were also documented. We obtained sociodemographic data including age, sex, and immunization status of patients. Patients were said to be appropriately immunised when they have received all the required doses of vaccines due for their age according to the Gambian National Programme on Immunization. Parental socioeconomic class was categorised according to the method described by Oyedeji [18]. This is based on occupation and highest level of educational qualifications of the parents. Gainfully employed professionals like doctors, engineers, architects etc. were classified as class one while the unemployed with no formal education were classified as class five [18]. Furthermore, the number of persons sleeping in the same room with the child was also noted and overcrowding was defined for this study as having three or more persons sleeping in the same room with the child [19].

 Study participants were examined to obtain their weight, height, or length that were used to derive the nutritional state according to the National Centre for Health Statistics and WHO (NCHS/WHO) weight-for-age, weight-for-height, and height-for-age parameters. Axillary temperature, respiratory rate, presence of pallor, central cyanosis, nasal flaring, head nodding, and altered sensorium at presentation were recorded. The respiratory rate was counted by observation when the child was quiet, calm, sleeping, or sucking using the WHO/UNICEF ARI counter over one minute, counting each upward movement of the abdomen and chest wall as one breath. Chest wall indrawing was identified as inward movement of the lower chest wall on breathing in when the child is lying flat on the mother's lap or on the examination couch. Central cyanosis was defined as bluish discoloration of the tongue and buccal mucosa, while impaired consciousness or altered sensorium was recorded as abnormal sleepiness or difficulty in waking up the child not responsive to verbal or painful stimulus. Abnormal breath sounds on auscultation as well as abnormal heart sounds were recorded.

 All patients enrolled into the study had a chest radiograph at admission which was read in digital format following the WHO guidelines for the interpretation of chest radiographs [20]. Enlarged heart (Cardiomegaly) was defined as cardiothoracic ratio of >0.55 in a well taken chest radiograph [21]. Also oxygen saturation (Osat) of the patients was measured using pulse oximeter (Nellcor N-200, USA) with appropriately sized paediatric probe attached to the finger or toe nail bed. The reading of the oximeter was recorded after stabilization of the reading for one minute by a trained nurse who was not involved in clinical examination. Hypoxaemia was defined as Osat <90%. Other investigations like Rapid Diagnostic Test for malaria using Optimal kit, full blood count including haemoglobin concentration, were done. 

 All the children were placed on intravenous antibiotics according to the unit's standard protocol for childhood pneumonia, fluid and calorie balance was ensured, oxygen therapy was administered when indicated, and children with associated congestive heart failure, pleural effusion, and other complications were appropriately treated. 

 Ethical clearance for the study was obtained from the Joint Gambian Government/MRC Ethical Committee. Informed consent was also obtained from parents/caregivers of each study participant.

### 2.1. Data Analysis

Data generated by the study were analyzed using Statistical Programme for Social Sciences (SPSS) software version 17.0 (SPSS Inc. standard version 2010). Means and standard deviations (SD) were determined for continuous variables while proportions and percentages were determined for categorical variables. Association between dependent (outcomes) and independent variables (predictors) was initially assessed by univariate analysis with the Pearson Chi square and the Fisher's exact test as appropriate (with Yate's correction where applicable). Level of significance at 95 percent confidence interval was taken at *P* < 0.05. Those that gave significant results in the univariate analysis were used in the stepwise multiple logistic regression analysis to determine their independent effect on dependent variables. Results were interpreted with odds ratios (OR) and 95 percent confidence interval (CI). Statistical significance was established when CI does not embrace unity.

## 3. Results

### 3.1. Study Participants

During the study period, we enrolled 420 (27.2%) children with severe pneumonia out of a total of 1517 under-five admissions. There was a slight male preponderance (male to female ratio was 1.2 : 1), 168 (40%) were infants, and mean (SD) age of the recruited patients was 18.0 (13.7) months (Table 1).

### 3.2. Socioeconomic Classification

The majority of the study patients 381 (90.7%) were from lower socioeconomic class with 370 (80.4%) in class IV and 11 (2.4%) in class V, while the remaining 39 (9.3%) were from the middle socioeconomic class III. No patient had parents in the upper socioeconomic classes I and II (Table 1).

### 3.3. Weight and Height of the Patients

The weight of the recruited patients ranged from 2.5 to 18.5 kg with a mean (SD) weight of 8.1 (2.6) kg. The mean (SD) height/length was 75.7 (12.1) cm, the median was 74.8 cm, and the height/length ranged from 49.8 cm to 110.0 cm.

### 3.4. Nutritional Status of the Recruited Patients

Of the 175 (41.7%) children who were undernourished, 89 (21.2%) had moderate wasting and 86 (20.5%) were severely wasted while nutritional oedema was present in 14 (3.3%) at presentation. One hundred and fifty (35.7%) were underweight while 71 children (16.9%) had features of chronic malnutrition manifesting as stunting (Table 1).

### 3.5. Associated Clinical Features

In addition to features of severe pneumonia, about 20.0% of the children had gastroenteritis, and 12.5% had features suggestive of heart failure.  Table 2 highlights other associated features seen in the children.

**Figure 1:**
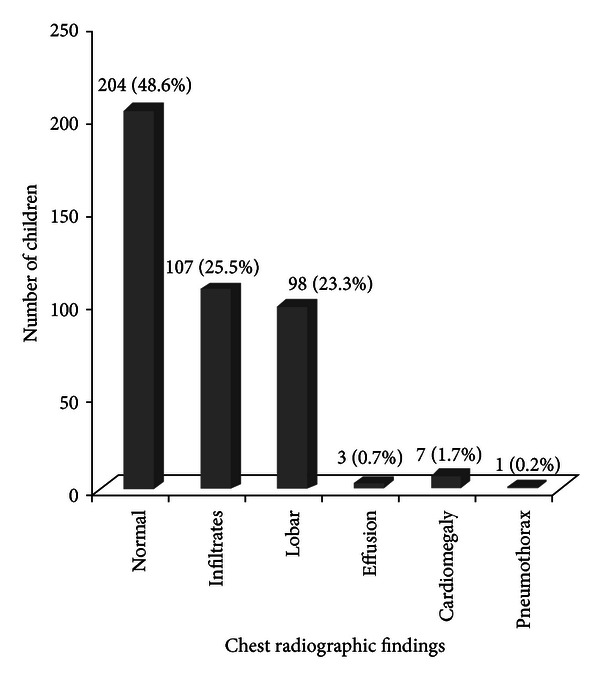
Chest radiographic findings of the 420 patients with severe pneumonia.

Chest radiograph was done on all enrolled patients and 48.6% of them were normal. The commonest abnormal finding was bilateral infiltrates followed by lobar consolidation (Figure 1).

### 3.6. Predictors of Hypoxaemia

 Eighty-one (19.3%) of the 420 patients were hypoxaemic at presentation. A greater proportion of infants was hypoxaemic at presentation compared to their older counterparts (24.5 versus 15.9%, *P* = 0.03) (Table 3). Grunting respiration, cyanosis, head nodding, and features of heart failure at presentation were also significantly associated with hypoxaemia. However, gender, increased respiratory rate, inability to suck/feed, convulsion, and nutritional state were not significantly associated with hypoxaemia (Tables 3 and 4). 

Further univariate analysis of laboratory parameters showed that only enlarged heart size on chest radiograph was significantly associated with hypoxaemia. Other laboratory parameters including anaemia, positive rapid malaria test, radiological pneumonia and bacterial isolates from blood or cerebrospinal fluid cultures were not significantly associated with hypoxaemia (Table 5).

The factors found to be significantly associated with hypoxaemia using univariate analysis (Tables 3 to 5) were subjected to stepwise multiple logistic regression analysis, and only cyanosis (OR = 83.200, 95% CI 5.248–355.111; *P* = 0.000) and grunting (OR = 5.210, 95% CI 2.287–7.482; *P* = 0.000) at presentation were significant independent predictors of hypoxaemia in children with severe pneumonia (Table 6).

## 4. Discussion 

 Hypoxaemia occurred in approximately 20% of the recruited patients in this study at presentation. This rate was higher than 4.0 and 5.9 percent reported by Usen et al. 1997 [9] and Usen and Weber 1999 [10], respectively, among Gambian children aged from 2 to 33 months with acute lower respiratory tract infections who presented to urban hospital setup. The much higher prevalence observed in the present study may be related to the fact that only severe cases of pneumonia were included unlike the previous studies where both nonsevere cases of acute respiratory tract infections, and reactive airway diseases like bronchial asthma were included. However, the proportion of hypoxaemic children reported in our study is close to the 25.6% reported among under-five children with WHO defined severe pneumonia in a large hospital in India [22]. Reports from high-altitude regions of Africa have higher proportions of hypoxaemia with pneumonia such as in 52% and 54%, respectively, from Kenya and Papua New Guinea [11, 12]. This may be related to the reduction of atmospheric oxygen pressure with increasing altitude or to the difference between the criteria used to select study subjects and define hypoxaemia. Nascimento-Carvalho et al. [23], however, reported a much lower prevalence of six percent among American infants with ARI and 10 percent among children with acute lower respiratory tract infections despite setting the cutoff of diagnosing hypoxaemia at <95 percent [23]. This may be due to early presentation of the patients to hospital where prompt and appropriate treatment was established before complications set in, as prevalence of hypoxaemia correlates well with increasing severity of ARI [23].

Our study has indicated that cyanosis and grunting respiration are independent predictors of hypoxaemia at presentation for severe pneumonia in children aged less than 5 years. This finding agrees with earlier studies from Gambia in which head nodding in addition to cyanosis and grunting were reported to be predictors of hypoxaemia [9, 10, 14]. Study from Nepal also reported that inability to feed as well as cyanosis and grunting respiration correlated well with hypoxaemia in children with severe pneumonia [24].

Grunting is an inspiratory breath sound produced when breathing in against a closed glottis; it is a form of positive pressure ventilation employed to overcome ventilation perfusion mismatch caused by consolidation or other causes of increased lung dead space [14]. Consequently, there will be a high proportion of deoxygenated haemoglobin which when in excess of 5 g/dL manifests clinically as cyanosis [14]. Cyanosis has been found to be a major determinant of the need for oxygen therapy in childhood pneumonia [9–12]. 

We conclude that one out of five children admitted with severe pneumonia may require oxygen therapy. Children admitted for pneumonia who are cyanosed and/or have grunting respiration should in addition to antibiotics be promptly commenced on oxygen therapy even if there is no means of confirming hypoxaemia.

## Figures and Tables

**Table 1:** Socio-demographic and general information of the patients.

Characteristics	Frequency	Percentage (%)
Age (in months)		
2–11	168	40
12–23	137	32.6
24–35	56	13.3
36–47	40	9.5
48–59	19	4.5
Total	**420**	**100.0**
Gender		
Male	232	55.2
Female	188	44.8
Total	**420**	**100.0**
Social class		
Class 1	0	0.0
Class 2	0	0.0
Class 3	39	9.3
Class 4	370	80.4
Class 5	11	2.4
Total	**420**	**100.0**
Appropriately immunised	340	81.9
Overcrowding	36	8.6
WHO/NCHS classification		
Weight for height		
Normal	245	58.3
Moderate wasting (*z* score < −2 SD)	89	21.2
Severe wasting (*z* score < −3 SD)	86	20.5
Total	**420**	**100.0**
Weight for age		
Normal	270	64.3
Underweight (*z* score < −2 SD)	150	35.7
Total	**420**	**100.0**
Height/length for age		
Normal	349	83.1
Stunted (*z* score < −2 SD)	71	16.9
Total	**420**	**100.0**

**Table 2:** Associated presenting features* among the 420 patients.

Clinical features at presentation	Frequency	Percentage (%)
Lethargy	13	3.1
Cyanosis	17	4.0
Convulsion	23	5.5
Inability to suck/feed	28	6.7
Features of heart failure	53	12.6
Head nodding	81	19.3
Vomiting	82	19.5
Diarrhoea	84	20.0
Grunting	126	30.0

*Some patients had multiple features at presentation.

**Table 3:** Association between Socio-demographic characteristics of the patients and the presence of hypoxaemia at presentation.

Socio-demographic characteristics	Hypoxaemic *n* (%)	Nonhypoxaemic *n* (%)	*P* value
Age range			
2–11	41 (50.6)	127 (37.5)	**0.030**
12–23	24 (29.6)	113 (33.3)	0.523
24–35	8 (9.9)	48 (14.2)	0.308
36–47	8 (9.9)	32 (9.4)	0.904
48–59	0 (0.0)	19 (5.6)	0.060**
Total	**81 (100.0)**	**339 (100.0)**	
Sex			
Male	39 (48.1)	193 (56.9)	0.153
Female	42 (51.9)	146 (43.1)	
Total	**81 (100.0)**	**339 (100.0)**	
Overcrowding	9 (11.1)	27 (8.0)	0.348
Not appropriately immunized	13 (16.0)	58 (17.1)	0.778

**Fisher's exact test applied; *n*: number of children. The figures in parentheses are percentages of the total in each column.

**Table 4:** Association between clinical features and presence of hypoxaemia at presentation.

Clinical features	Hypoxaemic *N* = 81 (%)	Nonhypoxaemic *N* = 339 (%)	*P* value
Duration of symptoms			
≤3 days	46 (56.8)	198 (58.4)	0.769
>3 days	35 (43.2)	140 (41.3)	
Total	**81 (100.0)**	**339 (100.0)**	
Gastroenteritis	16 (19.8)	68 (20.1)	1.000
*T* < 36.5°C	6 (7.4)	10 (2.9)	0.119*
Hyperpyrexia	6 (7.4)	19 (5.6)	0.538
RR ≥ 70 cpm	16 (19.8)	46 (13.6)	0.159
Grunting	49 (60.5)	77 (22.7)	**0.000**
Cyanosis	16 (19.8)	1 (0.3)	0.000**
Head nodding	28 (34.6)	53 (15.6)	**0.000**
Inability to suck/feed	7 (8.6)	21 (6.2)	0.428
Heart failure	21 (25.9)	32 (9.4)	**0.000**
Convulsion	2 (2.5)	21 (6.2)	0.185**
Somnolence/lethargy	1 (1.2)	12 (3.5)	0.472**
^ +^Underweight	28 (34.6)	122 (36.0)	0.811
^ +^Stunting	11 (13.5)	60 (17.7)	0.374
^ +^Severe wasting	18 (22.2)	68 (20.1)	0.665
^ !^Oedematous PEM	1 (1.2)	8 (2.4)	0.530**

*Yate's correction applied. **Fisher's exact test applied. ^+^WHO/NCHS classification: underweight = weight for age < −2 SD; stunting = height for age < −2 SD, while severe wasting = weight for height < −3 SD from the mean. The figures in parentheses are percentages of the total in each column. RR: respiratory rate, cpm: cycles per minute, and *T*: axillary temperature. ^!^Kwashiorkor and Marasmic kwashiorkor.

**Table 5:** Association of investigation findings and hypoxaemia at presentation.

Investigation results	Hypoxaemic *N* = 81 (%)	Nonhypoxaemic *N* = 339 (%)	*P* value
Severe anaemia	2 (2.5)	13 (3.8)	0.804**
Malaria (RDT)	5 (6.2)	30 (8.8)	0.434
Lobar opacities	24 (29.6)	74 (21.8)	0.136
Infiltrates	20 (24.7)	87 (25.7)	0.057
Cardiomegaly	4 (4.9)	3 (0.9)	0.038**
Bacteraemia	7 (8.6)	41 (12.1)	0.316
Meningitis	1 (1.2)	3 (0.9)	1.000**

**Fisher's exact test applied. The figures in parentheses are percentages of the total in each column.

**Table 6:** Predictors of hypoxaemia among the 420 patients with severe pneumonia.

Variable	*β*	SE	95% CI	OR	*P*
Age 2–11 months	0.473	0.304	0.881–2.921	1.711	0.122
Heart failure	−0.195	0.425	0.357–1.894	3.358	0.647
Head nodding	0.543	0.344	0.877–3.380	2.851	0.114
Grunting	1.420	0.305	2.287–7.482	5.210	**0.000**
Cyanosis	3.765	1.075	5.248–355.111	83.200	**0.000**
Cardiomegaly	−1.468	0.961	0.035–1.516	5.812	0.127

*β*: coefficient of regression; SE: standard error; 95% CI: confident interval; OR: odds ratio.
